# Serum Treatment and Its Limitations

**Published:** 1897-05-29

**Authors:** 


### Serum Treatment and Its Limitations.

The success which has attended the use of anti-toxin in diphtheria has led to the production of other forms of serum applicable to the treatment of diseases caused by a variety of micro-organisms. Of these the anti-streptococcus serum has taken the lead, many cases having been reported in which it has been used, apparently with considerable benefit, and now the cases in which the anti-pneumococcus serum has been employed in the treatment of pneumonia are beginning to come in. It may then be as well to point out how far we yet are from finality in this matter and how lacking in exactitude is much of our knowledge, especially in regard to the secondary infections by which death is caused in various admittedly microbial diseases. A few isolated observations point to there being some hope of the discovery at some time or other of a general anti-toxin. But so far as the practical application of bacteriology has hitherto gone, each form of pathogenic micro-organism seems to require a separate form of anti-toxin to control its effects, and herein lies the first great difficulty in the applica- of the treatment.

Diagnosis becomes all important. But bacteriological diagnosis takes time, and in view of the importance of applying the remedy early the time lost in making the diagnosis becomes a very serious matter.

A still more important cause of embarrassment, however, lies in the fact that the bacteriological diagnosis of the disease does not necessarily tell whether the fatal issue is threatened by the principal malady or by some superadded condition—some secondary infection. Mr. Bokenham, writing on the subject, properly enough insists on the importance of a definite diagnosis, pointing out that indiscriminate experiments with serum are unjustifiable. But the very fact that correct diagnosis is so important shows how liable one must be to error in those numerous cases in which the malady is due to a mixed infection. This is a difficulty which has been recognised from the first, even in regard to diphtheria, notwithstanding the fact that in that disease the diphtheria bacillus is the preponderating pathogenic influence. The uncertainty is still greater in diseases such as the so-called blood poisoning or septicæmia, in ulcerative endocarditis, and even in pneumonia.

In some of these conditions the microbes engaged are a more or less mixed lot even from the beginning, and in pneumonia, typhoid fever, whooping cough, and many other diseases, there is much reason for believing that although the original infection may be due to some one definite micro-organism, the thing that causes death may be something quite different, and in many cases not one organism but many.

Whooping-cough may, perhaps, be taken as the best example of the complexity of this problem. Suppose we were to isolate the infective micro-organism by which this disease is produced, and were to obtain an efficient anti-toxin by which its activity might be controlled, we should still be as far as ever from curing the fatal complications of the disease; for it is by the secondary infections rather than by the whooping-cough itself that death is caused, and when they have occurred the primary disease sinks into insignificance. Nevertheless, even in a compound morbid state, it seems probable that anything which will lessen the sum of the evil influences to which the body is exposed will to a certain extent do good, and so, to that extent, we may expect to gain by the use of anti-toxins, even in complex conditions, but we must not allow ourselves to be misled by the expectation of finding such diseases cut short by anti-toxins in the sudden and striking way which obtains in so many cases of diphtheria.

There is another perplexity to which we must allude. It is impossible to feel quite satisfied with a diagnosis as to the particular organism engaged which is arrived at by an examination of external discharges and exudations only. In certain cases practically pure cultures may be met with, and then, perhaps, all may be plain sailing; but when this is not the case it may be quite impossible, out of the various organisms found, to say which of them, or whether any one of them, is infecting the system, and which anti-toxin ought to be employed.

What we really want to know is which toxin is predominant, and is doing the mischief, and of ascertaining this by anything short of inoculation tests, tests applied to the blood itself, we do not see much hope at present. Hence the limitations to the utility of anti-toxins. There need, however, be no hesitation in using them when the clinical and bacteriological evidence points even to the probability of one or other of them being of service. When properly preprepared and carefully administered they seem to do no harm; while, on the other hand, the effect of their administration has in not a few instances been extremely striking, and one may fairly ask of what other remedy, offering such a probability of doing good, can it be said with equal positiveness that it will do no harm? We have endeavoured to indicate certain limitations to the utility of anti-toxins, but it by no means follows that corresponding limitations should be placed on their employment.

